# A peroxisome proliferator-activated receptor-δ agonist provides neuroprotection in the 1-methyl-4-phenyl-1,2,3,6-tetrahydropyridine model of Parkinson’s disease

**DOI:** 10.1016/j.neuroscience.2013.02.058

**Published:** 2013-06-14

**Authors:** H.L. Martin, R.B. Mounsey, K. Sathe, S. Mustafa, M.C. Nelson, R.M. Evans, P. Teismann

**Affiliations:** a Institute of Medical Sciences, University of Aberdeen, Aberdeen, United Kingdom; b Gene Expression Laboratory, Salk Institute, La Jolla, CA, USA

**Keywords:** DMEM, Dulbecco’s Modified Eagle Medium, DMSO, dimethyl sulfoxide, DPBS, Dulbecco’s phosphate-buffered saline, EDTA, ethylenediaminetetraacetic acid, FCS, foetal calf serum, GFAP, glial fibrillary acid protein, IL, interleukin, LDH, lactate dehydrogenase, MAC-1, macrophage antigen complex-1, MPP^+^, 1-methyl-4-phenylpyridinium iodide, MPTP, 1-methyl-4-phenyl-1,2,3,6-tetrahydropyridine, MTT, 3-(4,5-dimethylthizol-2-yl)-2,5-diphenyltetrazolium bromide, PBS, phosphate-buffered saline, PD, Parkinson’s disease, PFA, paraformaldehyde, PPAR, peroxisome proliferator-activated receptor, TH, tyrosine hydroxylase, TNFα, tumour necrosis factor-α, MPP^+^, neurodegeneration, neuroinflammation, SH-SY5Y, MPTP, PPAR delta

## Abstract

Peroxisome proliferator-activated receptor (PPAR)-γ and PPARα have shown neuroprotective effects in models of Parkinson’s disease (PD). The role of the third, more ubiquitous isoform PPARδ has not been fully explored. This study investigated the role of PPARδ in PD using 1-methyl-4-phenyl-1,2,3,6-tetrahydropyridine (MPTP) to model the dopaminergic neurodegeneration of PD. *In vitro* administration of the PPARδ antagonist GSK0660 (1 μM) increased the detrimental effect of 1-methyl-4-phenylpyridinium iodide (MPP^+^) on cell viability, which was reversed by co-treatment with agonist GW0742 (1 μM). GW0742 alone did not affect MPP^+^ toxicity. PPARδ was expressed in the nucleus of dopaminergic neurons and in astrocytes. Striatal PPARδ levels were increased (over two-fold) immediately after MPTP treatment (30 mg/kg for 5 consecutive days) compared to saline-treated mice. PPARδ heterozygous mice were not protected against MPTP toxicity. Intra-striatal infusion of GW0742 (84 μg/day) reduced the MPTP-induced loss of dopaminergic neurons (5036 ± 195) when compared to vehicle-infused mice (3953 ± 460). These results indicate that agonism of PPARδ provides protection against MPTP toxicity, in agreement with the effects of other PPAR agonists.

## Highlights

• We investigate the role of PPARδ in a model of Parkinson’s disease.
• PPARδ is upregulated after the neurotoxin MPTP.
• PPARδ antagonism enhances MPP^+^ toxicity which is reversible by PPARδ agonism.
• PPARδ agonism protects against MPTP-toxicity.

## Introduction

Parkinson’s disease (PD) is a common neurodegenerative disease (Dauer and Przedborski, 2003). Its primary neuropathogical feature is the loss of dopaminergic nigrostriatal neurons, which results in the disabling motor abnormalities that characterise PD: rigidity, bradykinesia, resting tremor and postural instability (Dauer and Przedborski, 2003). The pathogenesis of PD is poorly understood, but amongst the processes implicated in the degeneration of the dopaminergic neurons is inflammation, as evidenced by the activated glial cells and the upregulation of pro-inflammatory cytokines seen in both models of PD and PD patients (Czlonkowska et al., 1996; Hébert et al., 2003 McGeer et al., 1988; Mogi et al., 1994a,b).

Peroxisome proliferator-activated receptors (PPARs) are ligand-activated transcription factors with roles in fatty acid and carbohydrate metabolism (Desvergne and Wahli, 1999). There are three PPAR isoforms – α, γ and δ (also known as β), each with varying tissue distributions and ligand affinities (Desvergne and Wahli, 1999). In addition, PPARs have been shown to regulate inflammatory processes (Devchand et al., 1996; Ricote et al., 1999; Delerive et al., 2001). To date, the majority of the studies on the role of PPARs in neurodegenerative diseases have focussed on PPARγ, as it is known to be a negative regulator of macrophage, microglia and astrocyte function (Ricote et al., 1999; Storer et al., 2005; Xu et al., 2005, 2006). Indeed PPARγ agonists show neuroprotective effects in the 1-methyl-4-phenyl-1,2,3,6-tetrahydropyridine (MPTP) model of PD, with amelioration of MPTP-induced dopaminergic neuron loss and reduced gliosis (Breidert et al., 2002; Dehmer et al., 2004; Schintu et al., 2009; Martin et al., 2012). Similar neuroprotective effects have also been seen with the PPARα agonist fenofibrate (Kreisler et al., 2007).

In contrast to PPARα and PPARγ, less is known about the roles of the more ubiquitous PPARδ isoform, although the receptor is thought to have a function in inflammation control. Although these roles are less well understood, the general trend is towards anti-inflammatory action as PPARδ activation, like that of PPARγ, can inhibit the production of pro-inflammatory cytokines, such as tumour necrosis factor-α (TNFα), interleukin (IL)-1β and IL-6 (Bishop-Bailey and Bystrom, 2009). PPARδ can also control the inflammatory status of monocytes/macrophages (Bishop-Bailey and Bystrom, 2009). Indeed, PPARδ agonists have neuroprotective effects in models of Alzheimer’s disease and multiple sclerosis, which are concurrent with reduced glial cell activation (Niino et al., 2001; Escribano et al., 2009). This suggests that PPARδ activation could provide neuroprotection in PD. Furthermore, Iwashita et al. (2007) have shown that PPARδ agonists provide a degree of neuroprotection against both cerebral infarcts and MPTP, although the effects were not fully explored. Consequently, this study seeks to address the role of PPARδ in MPTP toxicity by using both an *in vivo* MPTP mouse model of PD and an *in vitro* model using 1-methyl-4-phenylpyridinium iodide (MPP^+^), the active metabolite of MPTP, in combination with the PPARδ agonist GW0742 and the PPARδ antagonist GSK0660.

## Experimental procedures

### Chemicals

GW0742 and GSK0660 were a kind gift of GlaxoSmithKline (Stevenage, UK). MPTP and MPP^+^ iodide were from Sigma–Aldrich, Poole, UK. All other chemicals unless otherwise stated were of analytical grade.

### Cell culture

Human neuroblastoma SH-SY5Y cells were cultured in Dulbecco’s Modified Eagle Medium (DMEM; Sigma–Aldrich) supplemented with 10% foetal calf serum (FCS; Biosera, Ringmer, East Sussex, UK) and 100 units/ml penicllin/streptomycin/glutamine (Invitrogen, Paisley, UK). Cells were kept at 37 °C in humidified 5% carbon dioxide and 95% air. Cells were seeded at 6000 cells/well in 96-well plates. All experiments were carried out 48 h after seeding and in serum-free media. GW0742 and GSK0660 were dissolved in dimethyl sulfoxide (DMSO) to make 1 mM solutions that were subsequently diluted with Dulbecco’s phosphate-buffered saline (DPBS; Sigma–Aldrich) and DMEM supplemented with 100 units/ml penicillin/streptomycin for experimental use. Final solutions contained 0.1% DMSO. MPP^+^ was dissolved in serum-free media and used at a final concentration of 1.5 μM. In experiments where GW0742 or GSK0660 was used together with MPP^+^, cells were pretreated with GW0742 or GSK0660 for 16 h before the addition of MPP^+^. In co-treatment experiments, cells were pretreated with GW0742 or GSK0660 as described above and the co-treatment was added at the same time as MPP^+^.

Mesencephalic dissociated neurons were prepared from the ventral mesencephalon of E14 rat (Sprague–Dawley) foetus as previously (Hsieh et al., 2011). Experimental protocols were in accordance with Home Office and institutional guidelines. The ventral mesencephalons from 15 embryos were collected in calcium- and magnesium-free Hank’s balanced salt solution (Invitrogen) containing 5 mM sodium bicarbonate (pH 7.0–7.2). Cells were dissociated with 0.25% trypsin in Hank’s balanced salt solution. Dissociation was stopped by the addition of an equal volume of foetal calf serum and 1 mg/ml DNAse (Roche). Thereafter, tissue was triturated three times with a wide pore, siliconised Pasteur pipette. Cells were plated on polyornithine and laminin-coated coverslips at a density of 2.5 × 10^5^ cells/cm^2^ in 24-well plates. Culture medium consisting of Dulbecco’s modified eagle medium with F12 nutrient mixture (Sigma) plus 1% N1 mix (Sigma), 10% FCS, 2 mM L-glutamine, 100 units/ml penicillin/streptomycin (Invitrogen), and 1 μg/ml insulin (Sigma) was supplied at 1000 μl/well.

Cells were maintained at 37 °C, 5% CO_2_ for 6 days. The culture medium was changed after 24 h and then changed every second day. Treatment was performed as described above with a final MPP^+^ concentration of 20 μM.

### Measurement of cell viability

Cell viability was determined by the conversion of the tetrazolium salt, 3-(4,5-dimethylthizol-2-yl)-2,5-diphenyltetrazolium bromide (MTT; Invitrogen) to its insoluble formazan. After treatment, 10 μl of MTT solution (5 mg/ml) was added to the plated cells and incubated at 37 °C for 4 h. Media were then removed and the formazan solubilised in 100 μl DMSO. The absorption of the resulting solution was measured at 570 nm with reference at 670 nm using a PowerWave XS microplate spectrophotometer (Bio-Tek, Potton, Bedfordshire, UK).

### Measurement of lactate dehydrogenase release

Release of lactate dehydrogenase (LDH) into the culture media from cells with damaged membranes was measured using an assay kit (Cayman Chemicals, Ann Arbor, MI) as per the manufacturer’s instructions.

### Apoptosis assay

The apoptosis assay was performed as described before (Hsieh et al., 2011). Apoptosis was detected by Hoechst 33258 staining (Molecular Probes). After immunocytochemistry staining, cells were incubated for 20 min with Hoechst 33258 (2 μg/ml). Healthy cells were identified by their evenly and uniformly stained nuclei. Apoptotic cells showed cell nuclear condensation and/or fragmentation. Apoptotic nuclei were counted as a percentage of total tyrosine hydroxylase (TH)-positive staining cells.

### Immunocytochemistry

Cells were fixed in 4% paraformaldehyde for 15 min. Cells were extensively washed with phosphate-buffered saline (PBS) between each step. Cells were permeabilised for 10 min in PBS containing 0.1% Triton-X (PBS-T). Unspecific binding was blocked with 10% normal goat serum (Vector Laboratories, Peterborough, UK) in PBS-T containing 0.3 M glycine. Cells were first incubated in primary antibodies [mouse tyrosine hydroxylase (1:200; Millipore, Watford, UK), PPARδ (1:100), Chemicon, Temecula, CA)] overnight at 4 °C before incubation in 1% normal goat serum in PBS-T with secondary antibodies [goat anti-mouse Cy3 (1:200; Jackson ImmunoResearch, West Grove, PA)] for 1 h at room temperature.

The coverslips were mounted, sealed and imaged by fluorescent microscopy at the same setting (LSM700, Carl Zeiss, Hertfordshire, UK).

### Animals and drug treatments

All procedures were in accordance with the Animals (Scientific Procedures) Act 1986 and MPTP handling and safety measures were consistent with Jackson-Lewis and Przedborski (2007). Twelve-week-old male C57BL/6 mice and PPARδ wild-type or heterozygote mice (previously described in Barak et al., 2002) received intraperitonal injections of MPTP–HCl (30 mg/kg free base) dissolved in saline, one injection for five consecutive days, and were sacrificed by decapitation at selected times ranging from 0 to 21 days after the last injection (3–7 mice per timepoint). Control mice received saline only.

GW0742 was dissolved in *N*,*N*-dimethylformamide (DMF; Fisher Scientific) and diluted with 0.1 M PBS. GW0742 does not readily cross the blood–brain barrier so for treatment with GW0742 intra-striatal infusion was used. Mice were anaesthetised with 120 mg/kg ketamine and 16 mg/kg xylazine. Once under anaesthesia, an L-shaped cannula was implanted into the right striatum at the following coordinates: 0.5 mm anterior to the bregma, 2 mm lateral to the midsagittal suture and 3 mm ventral to the skull. The cannula was connected to an Alzet osmotic pump (2002 model, Charles River, Margate, UK) to infuse either GW0742 or vehicle (25% DMF in 0.1 M PBS). Infusion rate was 0.5 μl/hour giving a total of 84 μg/day for 48 h prior to, throughout MPTP treatment (25 mg/kg free base for 5 consecutive days) and for 7 days afterwards. Analgesia (0.1 mg/kg buprenorphine) was given before surgery and on the day after surgery if necessary. Mice were sacrificed 21 days after the last MPTP injection and the implanted striatum dissected out and snap frozen on solid carbon dioxide. The remaining brain tissue was placed in 4% paraformaldehyde (PFA).

### Human samples

Human samples were obtained from the UK Parkinson’s Disease Society Tissue Bank at Imperial College, London. Selected PD and control samples were matched for age at death and interval from death to tissue processing. All procedures were approved by the responsible ethics committee (North of Scotland Research Ethics Committees).

### PPARδ, TH, glial fibrillary acid protein (GFAP), macrophage antigen complex-1 (MAC-1) and NeuN immunohistochemistry

This was performed as described in Teismann et al., 2003. Primary antibodies were mouse anti-PPARδ (1:250; Chemicon), rabbit anti-TH (1:500; Millipore), rabbit anti-GFAP (1:100; DAKO, Cambridgeshire, UK), rat anti-MAC-1 (1:100; Developmental Studies Hybridoma Bank, Iowa, USA) and rabbit anti-NeuN (1:100; Chemicon). Immunostaining was visualised with Alexa Fluor 488 anti-mouse (1:300; Molecular Probes, Eugene, OR) and cy-3 anti-rabbit (1:200; Jackson Immuno Research). Immunostaining was visualised by confocal microscopy (LSM 510, Carl Zeiss).

### RNA extraction and quantitative reverse transcriptase polymerase chain reaction (qRT-PCR)

Total RNA was extracted from selected brain regions using the TRIzol (Invitrogen) homogenisation method as in the manufacturer’s instructions. Samples were then subjected to a DNase digestion, DNase I Amp Grade kit (Invitrogen), and first strand cDNA synthesis was carried out using the Superscript II kit (Invitrogen). The primer sequences used in this study were PPARδ 5′-TAGAAGCCATCCAGGACACC-3′ (forward), 5′-CCGTCTTCTTTAGCC ACTGC-3′ (reverse), β-actin as 5′-TGTGATGGTGGGAATGGGTCAG-3′ (forward) and 5′-TTTGATGTCACGCACGATTTCC-3′ (reverse). Quantitative polymerase chain reaction amplification was undertaken using the Lightcycler 480 and the Lightcycler 480 SYBR green I Master (Roche Diagnostics, Lewes, UK) as in the manufacturer’s guidelines with an annealing temperature of 62 °C for PPARδ and 67 °C for β-actin. The identity of fragments amplified with these primers was confirmed by DNA sequencing performed by DNA Sequencing & Services (College of Life Sciences, University of Dundee, Scotland, www.dnaseq.co.uk) using Applied Biosystems Big-Dye Ver 3.1 chemistry on an Applied Biosystems model 3730 automated capillary DNA sequencer.

### Western blot analysis

Total proteins from mouse ventral midbrain, striatum and cerebellum samples were isolated in NP-40 buffer (20 mM Tris–HCl pH 8; 137 mM NaCl; 10% glycerol; 1% NP-40; 2 mM EDTA and protease inhibitors (cOmplete Mini EDTA-free cocktail, Roche)) 1:20 (wt/vol). Total proteins from human post-mortem ventral midbrain and striata were isolated in NP-40 buffer 1:5 (wt/vol). Protein concentration was determined using a bicinchoninic acid kit (Pierce, Rockford, IL). After boiling in Laemmli’s buffer, 20 μg of protein was separated by electrophoresis on a 10% sodium dodecyl sulphate–polyacrylamide gel, transferred to nitrocellulose membrane, and blocked with 5% non-fat dried milk in PBS containing 0.05% Tween-20 (vol/vol) for 1 h. Incubation with rabbit anti-PPARδ (1:1000; Alexis Biochemicals, San Diego, CA) or mouse anti-β-actin (1:25,000; Sigma–Aldrich) overnight at 4^o^C followed. Blots were then washed in PBS–Tween (0.05%) and incubated with either an anti-rabbit (1:5000) or anti-mouse (1:10,000) conjugated horseradish peroxidase antibody (Amersham Biosciences, Buckinghamshire, UK) at room temperature for 1 h. Blots were then washed in PBS–Tween (0.05%) and developed using a chemiluminescence solution (1 ml (50 mg luminol sodium salt (Sigma–Aldrich) in 200 ml 0.1 M Tris–HCl pH 8.6), 100 μl (11 mg *p*-coumaric acid (Sigma–Aldrich) in 10 ml DMSO) and 0.3 μl 30% hydrogen peroxide). Bands were visualised with an AlphaInnotech digital imaging system (San Leandro, CA) and quantified with AlphaEase FC 5.02 software.

### Stereological counting and analysis of striatal TH-immunoreactivity

Immunostaining for stereological counting of TH and Nissl-stained substantia nigra pars compacta (SNpc) neurons was carried out on midbrain sections as described in Wu et al. (2002). Every fourth section was taken until there were 12 sections for each SNpc. The primary antibody was a polyclonal rabbit anti-TH (1:1000; Millipore) and staining was visualised with 3,3′-diaminobenzidine (Sigma–Aldrich). The sections were counted using regular light microscopy (AxioImager M1, Carl Zeiss) and the optical fractionator method (West, 1993) (Stereo Investigator version 7, MBF Bioscience, Magdeburg, Germany).

For analysis of striatal TH-immunoreactivity, every eighth section of the striatum stained as described (Wu et al., 2002) (rabbit anti-TH (1:500; Millipore)). TH-immunoreactivity was assessed on scans (Hewlett Packard Scanjet G3110, Bracknell, Berkshire, UK) of the sections using Scion Image (Version 4.0.3.2 Scion Corporation, MD).

### HPLC analysis of striatal dopamine and 3,4-dihydroxyphenylacetic acid (DOPAC) levels

High-performance liquid chromatography (HPLC) with electrochemical detection was used to measure striatal levels of dopamine and DOPAC using a method that has been described (Nuber et al., 2008). Briefly, mice were killed 21 days after the last MPTP injection and the striata were dissected out and snap frozen on solid carbon dioxide. Striata were then homogenised in 0.1 M perchloric acid (1:30 wt/vol), sonicated and centrifuged at 18,600*g* at 4 °C for 20 min. Following centrifugation, 20 μl of sample was injected onto a C18 column (Dionex, Germering, Germany) The mobile phase consisted of 90% 50 mM sodium acetate, 35 mM citric acid, 105 mg/L octane sulfonic acid, 48 mg/L sodium EDTA solution and 10% methanol at pH 4.3 methanol. Flow rate was 1 ml/min. Peaks were detected by an ESA Coulchem II electrochemical detector (ESA, Dionex) and the detector potential was set at 700 mV. Data were collected and processed using the Chromeleon computer system (Dionex).

### Striatal MPP^+^ levels

Liquid chromatography with on-line ultraviolet detection/tandem mass spectrometry (LC–UV–MS–MS) was used to measure striatal levels of MPP^+^. Briefly, mice received drug treatment as outlined in Section ‘Apoptosis assay’ and, 90 min after a single MPTP injection (25 mg/kg), mice were sacrificed. The implanted striata were dissected out and snap frozen on solid carbon dioxide. Striata were then sonicated in 0.1 M perchloric acid (1:30 wt/vol) and centrifuged at 14,000 rpm (18,620*g*; Mikro 200R) at 4 °C for 20 min. Following centrifugation, 2 μl of sample was injected onto a Hichrom 5 μ C18 column (Hichrom, Theale, UK). The mobile phase consisted of 80% 0.1% formic acid in water/20% 0.1% formic acid in acetonitrile. Flow rate was 200 μl/min. MPP^+^ was detected by a photodiode array detector set at 295 nm and a triple quadrupole mass spectrometry with a mass to charge ratio of 170–128 at 32 V and 1.9 m Torr (ThermoSurveyor PDA/TSQ Quantum, ThermoScientific, Loughborough, UK). Data were collected and processed using Xcalibur 2.0.7 SP1.

### Statistical analysis

Data was analysed in SigmaPlot 11 for Windows (Systat Software Inc., Chicago, IL). All values are expressed as the mean ± SEM. Normal distribution of the data was tested and the homogeneity of variance confirmed with Levene Test. For single pairs of data Student *t*-tests were used for comparisons between means. For data sets greater that single pairs analysis of variance (ANOVA) was used to analyse differences among means with time, treatment, or genotype as the independent factor, when the data were normally distributed. When ANOVA showed significant differences post hoc testing was used to make comparisons between means, Dunnett’s post hoc test was used for time-course studies and Student–Newman–Keuls was used to make pairwise comparisons in all other studies. Data not normally distributed were analysed with the Kruskal–Wallis test followed by Mann–Whitney U-tests. The null hypothesis was rejected at the 0.05 level.

## Results

### Impacts of a PPARδ agonist and antagonist *in vitro* on MPP^+^-induced cytotoxicity

**Fig. 1:**
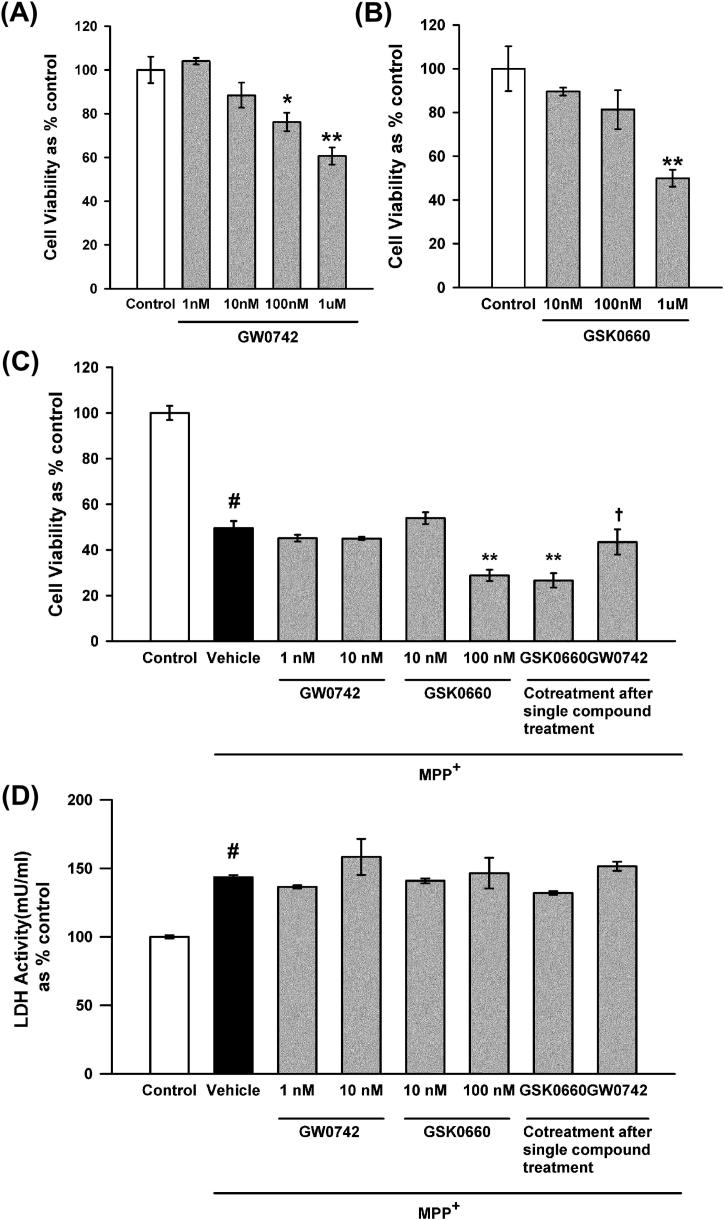
Effects of the PPARδ agonist GW0742 and the antagonist GSK0660 on MPP^+^ cytotoxicity as measured by cell viability and LDH release. The impact of GW0742 (A) and GSK0660 (B) on cell viability was assessed by MTT reduction. Concentrations of GW0742 above 10 nM and concentrations of GSK0660 above 100 nM decreased cell viability compared to control (0.1% DMSO). GSK0660 (100 nM) and GSK0660 pre-treatment followed by co-treatment with GW0742 (10 nM) increased the MPP^+^-induced decrease in cell viability compared to MPP^+^ alone (C). This was reversed by pre-treatment with GW0742 (10 nM) and then co-treatment. Neither GW0742 nor GSK0660 affected MPP^+^ induced LDH release (D). Data are mean ± SEM, *n* = 3, ^#^*p* < 0.05 MPP^+^ compared to control; ^∗^*p* < 0.05; ^∗∗^*p* < 0.01 compared to MPP^+^ alone, ^†^GW0742 pre-treatment compared to GSK0660 pre-treatment (ANOVA followed by Student–Newman–Keuls post hoc test).

The effects of the PPARδ agonist GW0742 and the antagonist GSK0660 on MPP^+^-induced cytotoxicity in SH-SY5Y cells, a dopaminergic neuroblastoma cell line, were investigated. These compounds have a high affinity for PPARδ over the other PPAR isoforms, demonstrating a selectivity of over 1000-fold for PPARδ (Table 1). Both GW0742 and GSK0660 decreased cell viability compared to solvent-only treatment at concentrations above 100 nM for GW0742 (*p* = 0.017 ANOVA, Student–Newman–Keuls post hoc test; Fig. 1A) and above 1 μM for GSK0660 (*p* = 0.005 ANOVA, Student–Newman–Keuls post hoc test; Fig. 1B). Subsequently, the impacts of these compounds on MPP^+^-induced cytotoxicity were assessed using maximum concentrations of 10 nM for GW0742 and 100 nM for GSK0660. The cytotoxicity of MPP^+^ was unaffected by treatment with GW0742, but was increased in the presence of 100 nM GSK0660 as measured by a reduction in cell viability compared to MPP^+^ alone (*p* = 0.008 ANOVA, Student–Newman–Keuls post hoc test; Fig. 1C). This increase in toxicity was reduced by pre-treatment with GW0742 and subsequent co-treatment with GSK0660, and was therefore due to a pharmacological effect of GSK0660, and not due to any synergistic toxic effects with MPP^+^. Co-treatment following GSK0660 pre-treatment did not affect the increase in toxicity compared to MPP^+^ alone. Despite these alterations in cell viability neither 100 nM GSK0660, 10 nM GW0742 or the co-treatments had any effects on MPP^+^-induced cytotoxicity as measured by LDH release (Fig. 1D), suggesting that inhibition of PPARδ may affect cellular metabolic status, altering MTT conversion to its insoluble formazan, although this does not lead to an alteration in cell death in this model.

**Fig. 2:**
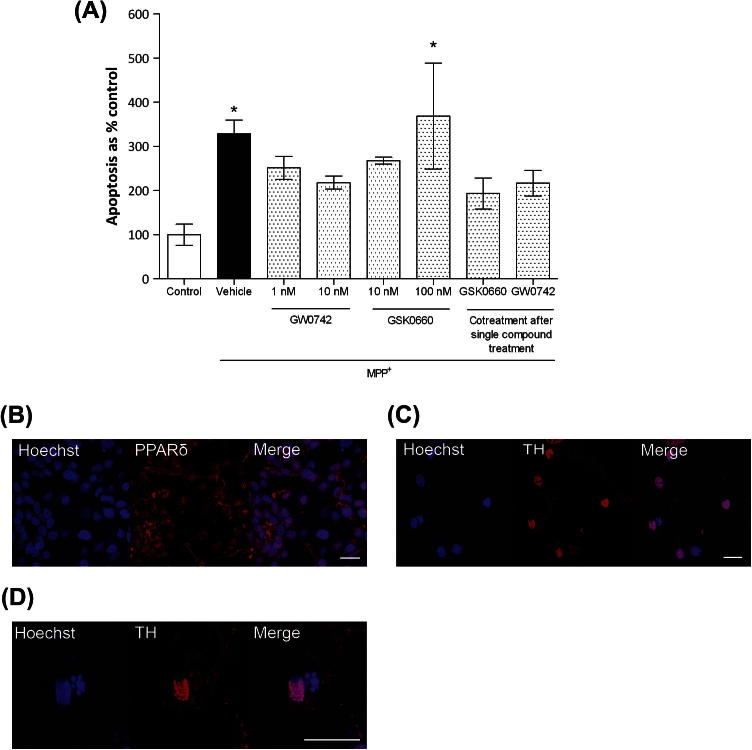
Effect of the PPARδ agonist GW0742 and antagonist GSK0660 on apoptosis in rat ventral midbrain dopaminergic cells. MPP^+^ (20 μm) administration caused an increase in cellular apoptosis as compared to control (0.1% DMSO) cells. Addition of 100 nM GSK0660 exacerbated this effect. No effect was evident with GW0742 administration or co-treatments (A). Nuclear (blue) co-localisation of PPARδ (red) in ventral midbrain dopaminergic cells (B). Imaging of MPP^+^-treated cells, blue nuclear staining with Hoechst 33258 and red TH-immunoreactivity (C). Apoptotic cells were identified by nuclear condensation and/or fragmentation (D). Data are mean ± SEM. All treatments were performed in triplicate and the average taken from four independent experiments. The results were compared by one-way ANOVA and Newman–Keuls post hoc test. ^∗^*p* < 0.05 compared to control. (TH – tyrosine hydroxylase) Scale bars = 20 μm. (For interpretation of the references to colour in this figure legend, the reader is referred to the web version of this article.)

Apoptotic cell counts using the same treatment regimen in primary dopaminergic neurons showed that co-treatments with either GW0742 and/or GSK0660 had no effect on MPP^+^-induced cytotoxicity (Fig. 2A) as determined by apoptotic cell counts. Although not significant, GW0742 showed a tendency to protect against MPP^+^-induced toxicity and ameliorate the additive effects of GSK0660 on MPP^+^-induced toxicity.

### Effects of MPTP treatment on PPARδ expression *in vivo*

**Fig. 3:**
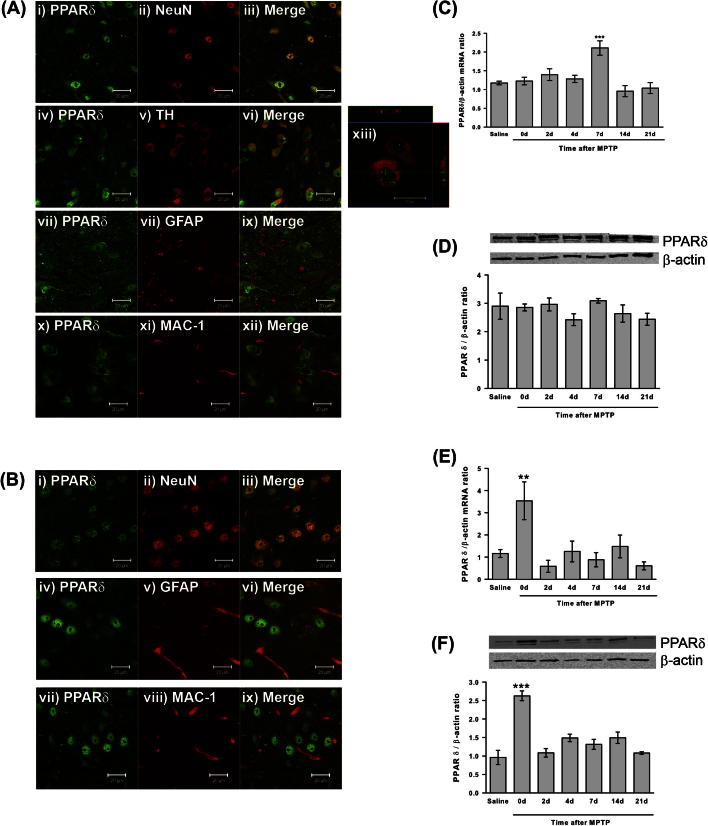
PPARδ immunolocalisation and alterations in PPARδ expression following MPTP treatment. Double immunofluorescence confirms that 2 days after MPTP treatment PPARδ (green) is expressed in neuronal nuclei in the substantia nigra (A) and striatum (B) labelled with NeuN (i–iii; red), including in TH-positive neurons ((A) iv–vi and xiii; red), and in GFAP-positive astrocytes ((A) vii–ix and (B) iv–vi; red). PPARδ was not expressed in MAC-1-positive microglia ((A) x–xii and (B) vii–ix; red). PPARδ mRNA levels in the ventral midbrain are increased 7 days after MPTP compared to saline-treated mice (A), but no alterations in PPARδ protein levels are seen in this region after MPTP (B). In the striatum mRNA (C) and protein (D) PPARδ levels are increased immediately after MPTP before returning to basal levels. PPARδ protein levels are unchanged in the cerebellum after MPTP treatment (E). Data are mean ± SEM, *n* = 3–6 mice per timepoint. ^∗∗^*p* < 0.01, ^∗∗∗^*p* < 0.001 compared to saline (ANOVA with Dunnett’s post hoc test) (d – days after MPTP (5 × 30 mg/kg) administration) (TH – tyrosine hydroxylase; GFAP – glial fibrillary acidic protein; MAC-1 – macrophage antigen complex-1). Scale bars = 20 μm. (For interpretation of the references to colour in this figure legend, the reader is referred to the web version of this article.)

Having ascertained that inhibition of PPARδ activation impacts on MPP^+^ cytotoxicity in a cell culture model of PD the next step was to determine the immunohistological localisation of PPARδ *in vivo*. This was examined by fluorescent double-labelling using TH as a marker for dopaminergic cells, GFAP as a marker for astrocytes, MAC-1 as a marker for microglia and NeuN as a general neuronal marker two days after MPTP treatment. PPARδ is widely expressed in neuronal nuclei in both the SNpc and the striatum (Fig. 3A i–iii and B i–iii), including the nuclei of TH-positive cells in the SNpc (Fig. 3A iv–vi and xiii). PPARδ also co-localised with GFAP, indicating expression in astrocytes in both the SNpc and the striatum (Fig. 3A viii–ix and B iv–vi). No expression of PPARδ was detected in microglia (Fig. 3A x–xii and B vii–ix). Following this confirmation that PPARδ is expressed in the SNpc and striatum, the impact of MPTP treatment on PPARδ levels was determined. Quantitative PCR showed a significant increase in PPARδ mRNA in the ventral midbrain (the area containing the SNpc) 7 days after MPTP administration compared to saline-treated mice (*p* < 0.001 ANOVA, Dunnett’s post hoc test; Fig. 3C) when normalised to β-actin levels (β-actin levels were unchanged by MPTP treatment, data not shown). PPARδ mRNA levels were also transiently increased in the striatum, where the dopaminergic neurons of the SNpc terminate. In contrast to the ventral midbrain, this increase was immediately after MPTP treatment (*p* = 0.011 ANOVA, Dunnett’s post hoc test; Fig. 3D). Western blot analysis was used to confirm these changes at the protein level. Interestingly, levels of PPARδ protein in the ventral midbrain were unaffected by MPTP treatment (Fig. 3E), as was the case for cerebellum, a control tissue (data not shown). In the striatum, the level of PPARδ protein was significantly increased immediately after MPTP treatment (*p* < 0.001 ANOVA, Dunnett’s post hoc test; Fig. 3F), which correlated with the increase in PPARδ mRNA levels.

### Genetic manipulation of PPARδ levels does not alter MPTP toxicity

**Fig. 4:**
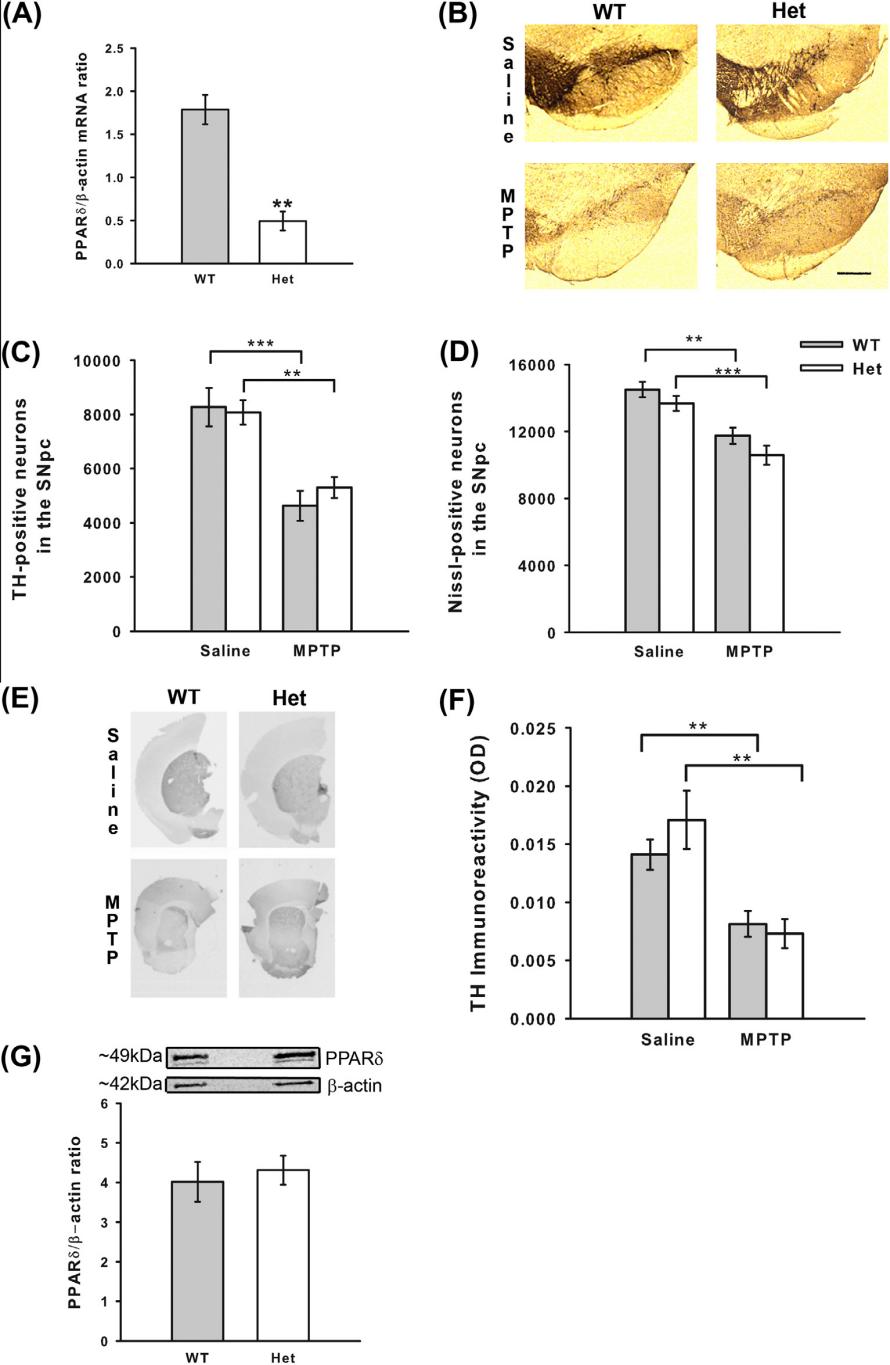
Effects of genetic manipulation of PPARδ levels on MPTP neurotoxicity. PPARδ heterozygous mice have reduced PPARδ mRNA levels compared to their wild-type littermates (A). No difference is seen between wild-type mice and their heterozygous littermates (null mice were not viable) in their sensitivity to MPTP toxicity. Representative micrographs of TH- and Nissl-stained sections (B) (Scale bar = 200 μm). Both TH-positive neuron (C) and Nissl-positive neuron (D) numbers were reduced by MPTP in wild-type and heterozygous mice. No differences were detected in striatal TH-immunoreactivity (E and F) between wild-type and heterozygous mice. PPARδ protein levels in untreated mice were not significantly different between heterozygous mice and their wild-type littermates (G). Data are mean ± SEM, *n* = 6–7 mice per group for stereological counting and *n* = 3 mice per group for mRNA and protein analysis. ^∗∗^*p* < 0.01; ^∗∗∗^*p* < 0.001 (Student *t*-test (A) or ANOVA with Student–Newman–Keuls post hoc test) (WT – wild-type; Het – heterozygous; TH – tyrosine hydroxylase; SNpc – substantia nigra pars compacta).

Having established that PPARδ levels are altered by MPTP treatment and that GSK0660 increased MPP^+^ cytotoxicity *in vitro*, the effects of reducing PPARδ levels *in vivo* on MPTP toxicity were explored. Due to the low bioavailability of GSK0660 (Shearer et al., 2008), a genetic approach was attempted, however, mice homozygous-null for PPARδ are not viable due to ectoplacental defects (Barak et al., 2002; Wang et al., 2007). A comparison of PPARδ mRNA levels in untreated heterozygous mice and their wild-type littermates was undertaken to ensure significant reductions in PPARδ expression. PPARδ mRNA in heterozygous mice was reduced by approximately 70% (*p* = 0.003 Student *t*-test; Fig. 4A). The response of heterozygous mice and their wild-type littermates to MPTP was then assessed and there were no differences in their sensitivity to MPTP-induced neuron loss (Fig. 4B–D). MPTP reduced both TH-positive and Nissl-positive neuron numbers when compared to saline-treated mice of the appropriate genotype (*p* < 0.001 ANOVA with Student–Newman–Keuls post hoc test). Striatal TH-immunoreactivity was also assessed for differences between wild-type and heterozygous mice in response to MPTP treatment, although no differences were observed (Fig. 4E, F). The levels of dopamine and DOPAC, a major metabolite of dopamine, in the striatum were reduced by MPTP treatment in both wild-type and heterozygous mice, as measured by HPLC (*p* < 0.001 Kruskal–Wallis test with Mann–Whitney U-post hoc tests; Table 2) Following the lack of impact of genetic manipulation on MPTP toxicity, the levels of PPARδ protein between wild-type and heterozygous mice were examined in untreated mice. In contrast to PPARδ mRNA levels, there was no significant reduction in PPARδ protein in heterozygous mice compared to their wild-type littermates (Fig. 4G), which may underlie the lack of alteration in sensitivity to MPTP treatment in these mice.

### Treatment with the PPARδ agonist GW0742 provides neuroprotection against MPTP toxicity

**Fig. 5:**
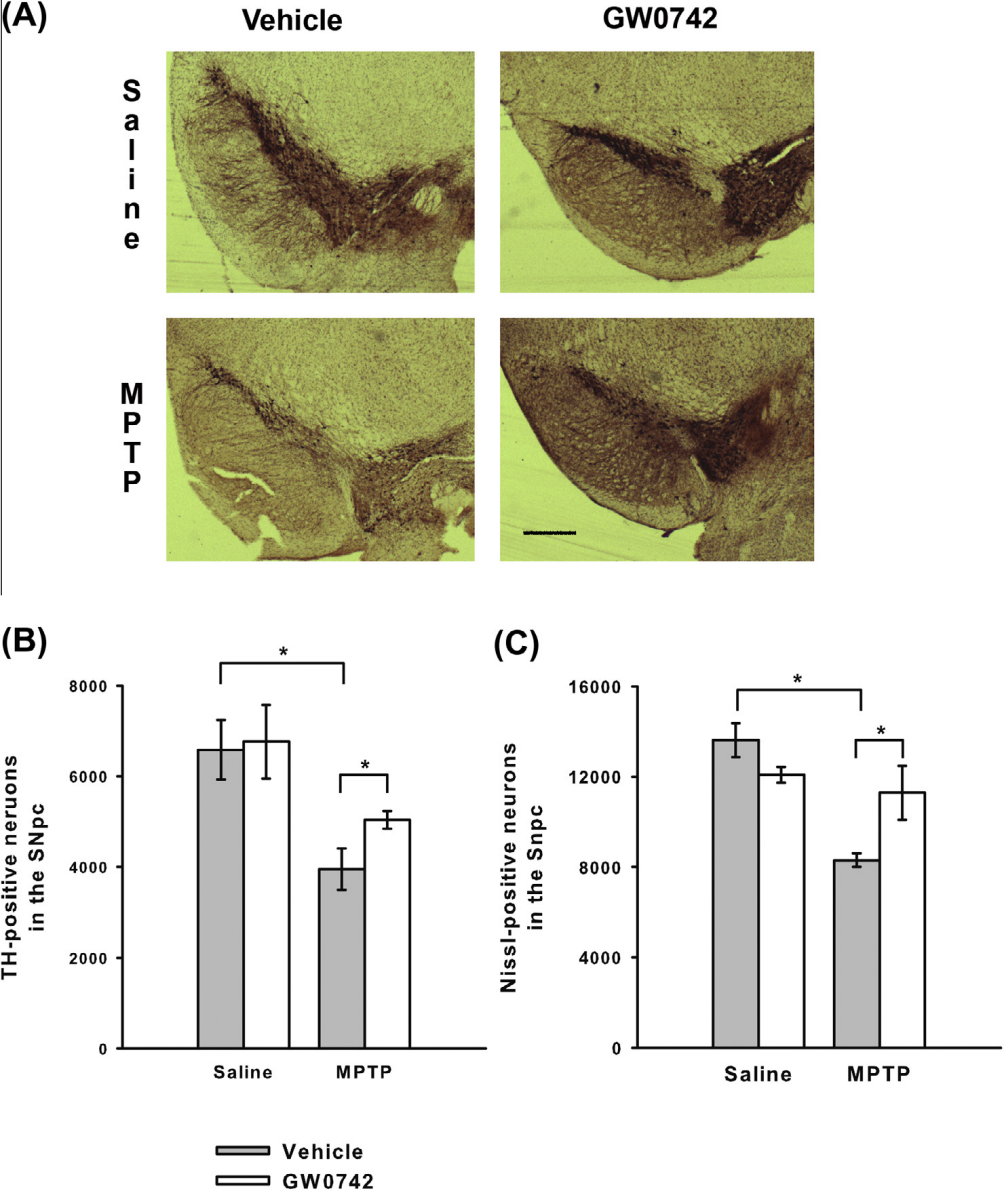
Effects of intra-striatal infusion of the PPARδ agonist GW0742 on MPTP neurotoxicity. (A) Representative micrographs of TH and Nissl-stained sections following infusion with GW0742 or vehicle (25% DMF in PBS) (Scale bar = 200 μm). Infusion of GW0742 reduced MPTP-induced loss of TH-positive (B) and Nissl-positive (C) neurons compared to infusion of vehicle. Data are mean ± SEM, *n* = 3–5 mice per group. ^∗^*p* < 0.05 (ANOVA with Student–Newman–Keuls post hoc test; Nissl – Kruskal–Wallis test with Mann–Whitney U-post hoc test) (TH – tyrosine hydroxylase; SNpc – substantia nigra pars compacta).

The data from the PPARδ heterozygous mice were not definitive, as these mice had the same expression level of PPARδ protein as their wild-type littermates. Subsequently, pharmacological modulation of PPARδ with intra-striatal infusion of the agonist GW0742 was undertaken, as this had reversed the effects of GSK0660 *in vitro*. Infusion of GW0742 into the striatum was chosen since this was the region where consistent alterations in PPARδ levels following MPTP treatment were observed. GW0742 infusion did not affect MPTP-induced decreases in dopamine and its metabolites in the striatum (Table 3). However, GW0742 infusion did reduce MPTP-induced decreases in TH-positive and Nissl-positive neuron numbers in the SNpc compared to mice infused with vehicle only (TH *p* = 0.044 ANOVA, Student–Newman–Keuls post hoc test; Nissl *p* = 0.036 Kruskal–Wallis test with Mann–Whitney U-post hoc tests; Fig. 5). This protection was not due to alterations in MPTP bioactivation to MPP^+^ as striatal levels of MPP^+^ were greater in the mice receiving GW0742 than in the mice receiving vehicle only (Table 4).

### Human Parkinson’s disease patients show no changes in PPARδ levels

**Fig. 6:**
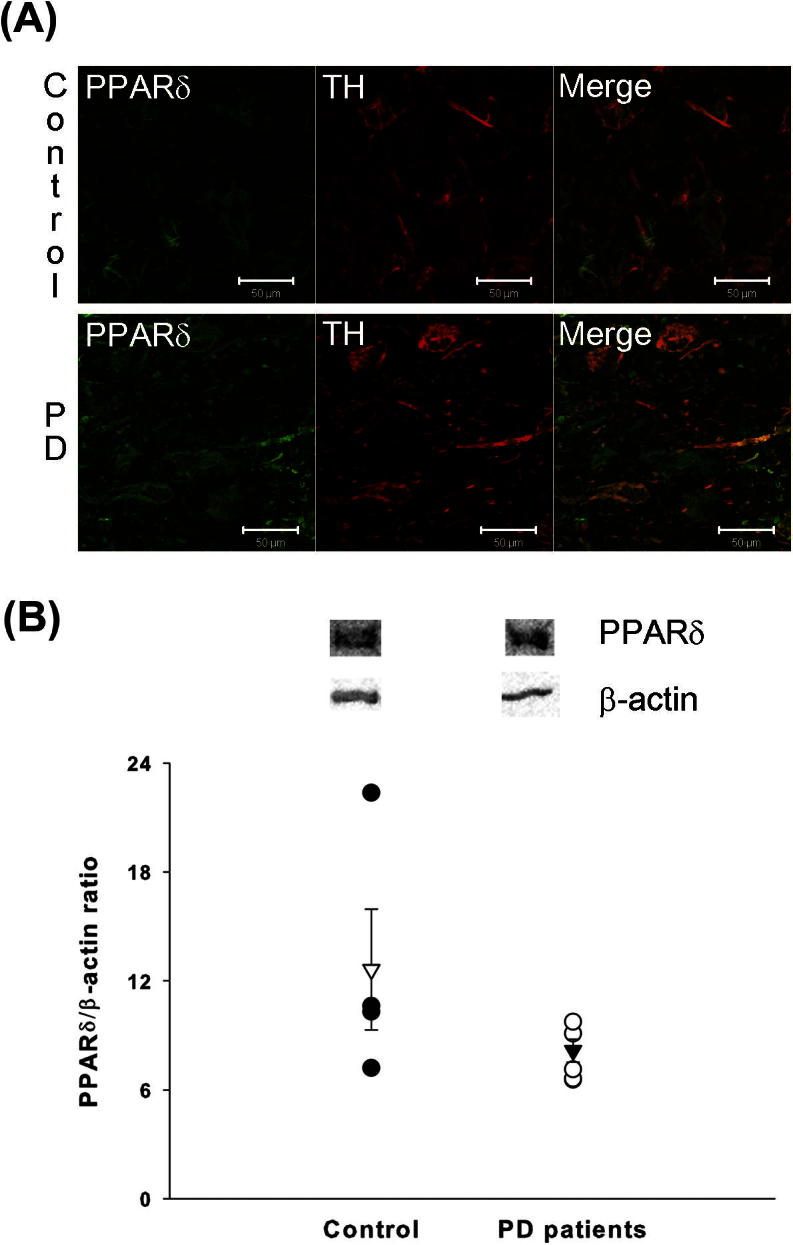
PPARδ in human post-mortem tissue. Double immunofluorscence confirms that PPARδ (green) is expressed in dopaminergic neurons (TH-positive; red) in the substantia nigra ((A) i–iii). No difference in PPARδ protein level in the ventral midbrain is seen between PD patients and controls (B). Open triangle is mean ± SEM for control; Closed triangle is mean ± SEM for PD patients; *n* = 4–6. (PD – Parkinson’s disease; TH – tyrosine hydroxylase) Scale bars = 50 μm. (For interpretation of the references to colour in this figure legend, the reader is referred to the web version of this article.)

To investigate possible changes in PPARδ levels in PD, its expression in post-mortem tissue from PD patients was assessed. Firstly, the localisation of PPARδ in the SNpc of PD patients was established. PPARδ was consistently expressed in TH-positive neurons within the SNpc, correlating with the findings in SH-SY5Y cells and those in mice (Fig. 6A). Having determined that PPARδ was expressed in PD patients, Western blot analysis was performed to ascertain whether any alterations in PPARδ protein levels could be detected compared to control tissue. No alterations in PPARδ protein levels were observed between the ventral midbrains of PD patients and controls (Fig. 6B), consistent with the results from the mouse study. PPARδ protein was not detected in the striatum of either PD patients or controls.

## Discussion

This study sought to determine the role of PPARδ in MPTP toxicity, as activation of the other PPAR isoforms show neuroprotective effects (Breidert et al., 2002; Dehmer et al., 2004; Kreisler et al., 2007; Schintu et al., 2009; Martin et al., 2012). Intra-striatal infusion of GW0742 was neuroprotective *in vivo* against MPTP-induced dopaminergic neuron loss. This protective effect of GW0742 did not extend into the striatum despite this being the region where consistent changes in PPARδ levels were seen. This is in contrast to the work of Iwashita et al. (2007), who saw an attenuation of the MPTP-induced decreases in striatal dopamine and DOPAC levels following intra-cerebral ventricular infusion with two other PPARδ agonists, L-165041 and GW501516. The effects on dopaminergic neuron number were not assessed. The differences between the work of Iwashita et al. (2007) and this study may arise from variations in the MPTP regimes, infusion site and doses of agonist used. Indeed the protective effects of L-165041 and GW501516 were only seen with doses of 120 μg/day, which is higher than the dose used in this study (84 μg/day).

The neuroprotective effects of GW0742 were not seen *in vitro* in SH-SY5Y cells, although treatment with GW0742 attenuated the detrimental effects of GSK0660 treatment on MPP^+^-cytotoxicity. It is likely that these discrepancies are the result of PPARδ being expressed in both astrocytes and neurons *in vivo* compared with neuronal cells only *in vitro*. Indeed, PPARδ expression after MPTP treatment was upregulated in the striatum in a time-frame that was compatible with that of astrogliosis (Ciesielska et al., 2009). Astrocytes, together with microglia, are an important source of both pro- and anti-inflammatory mediators including TNFα, IL-6 and IL-10 (Dong and Benveniste, 2001; Long-Smith et al., 2009), and the other PPAR isoforms are documented to have anti-inflammatory effects. Moreover, agonists of both PPARα and PPARγ are known to reduce nitric oxide and pro-inflammatory cytokine release from activated microglia and astrocytes (Dehmer et al., 2004; Santos et al., 2005; Storer et al., 2005; Xu et al., 2006; Nicolakakis et al., 2008; Yi et al., 2008; Escribano et al., 2009). These anti-inflammatory mechanisms are thought to underlie the neuroprotective effects of PPARα and PPARγ agonists against MPTP toxicity (Breidert et al., 2002; Dehmer et al., 2004; Kreisler et al., 2007; Schintu et al., 2009). Therefore it is possible that the protective effects of GW0742 are mediated by anti-inflammatory mechanisms potentially focussed on astrocytes, as no expression of PPARδ was detected in microglia. This is supported by the lack of effect of GW0742 against MPP^+^ toxicity *in vitro*. Whether these anti-inflammatory actions are direct or result from the release of transcriptional repression of the other PPAR isoforms is not clear, as non-liganded PPARδ inhibits the ligand-induced transcriptional activity of other PPAR isoforms (Shi et al., 2002). Further exploration of the effects of PPARδ agonists *in vivo* should seek to clarify if the protective effects of GW0742 are PPARδ-dependent and if these effects are mediated by an alteration of the inflammatory responses generated by MPTP treatment.

*In vitro* data where GSK0660 reduced cell viability, an effect reversed upon co-treatment with GW0742, suggest that a degree of basal activity of PPARδ is required to maintain neuronal cell viability. Indeed, GSK0660 has been reported to act as an inverse agonist when administered alone (Shearer et al., 2008) and PPARδ is important in cellular metabolic pathways (Basu-Modak et al., 1999; Luquet et al., 2005). This is further supported by the maintenance of wild-type levels of PPARδ protein seen in PPARδ heterozygous mice, even though these mice had approximately half the level of PPARδ mRNA compared to their wild-type littermates. This type of discrepancy between mRNA and protein levels has been reported in mice heterozygous for other genes (Chen et al., 1997; Takahashi et al., 2002), and could be expected if PPARδ has a significant and necessary function in the basal activity in neurons. The nature of this potential basal activity is currently unclear.

In the ventral midbrain only PPARδ mRNA levels were upregulated. The lack of a concurrent protein upregulation is not unusual, as increases in mRNA levels do not always correlate with increases in protein levels (Chen et al., 2002; Pascal et al., 2008) and activation of mouse liver PPARα and PPARγ with Wy-14643 and rosiglitazone, respectively, only gave a 40% correlation between changes in mRNA and protein levels (Tian et al., 2004). The lack of alteration in PPARδ protein levels in the ventral midbrain was reflected in human post-mortem tissue when compared to control tissue, suggesting that there may be a degree of correlation between the mouse model and the clinical situation. Unfortunately, PPARδ protein was not detected in the human striatal extracts and, to the authors’ knowledge, PPARδ has not yet been detected in human striatum elsewhere in the literature. Species differences in PPARδ expression between human and rodent tissues have been reported in urothelium and intrafollicular epidermal cells (Chopra et al., 2008; Yacoub et al., 2008). This means that the degree of correlation between PPARδ expression in MPTP toxicity and in PD pathogenesis remains unclear. However, changes between PD patients and control tissue may not have been seen since PPARδ levels were only transiently increased in mouse striatum immediately after MPTP, while the human post-mortem samples represent a later stage of disease progression.

## Conclusion

This study shows that GW0742 provides neuroprotective effects in a mouse model of PD, which supports findings from other neurodegenerative diseases including multiple sclerosis and Alzheimer’s disease (Polak et al., 2005; Kalinin et al., 2009). As the precise functions of PPARδ in neurons and astrocytes have not been delineated, the cellular mechanisms underlying these protective effects remain unclear. The importance of the PPARδ basal activity suggested by the *in vitro* work indicates that the protective effects of GW0742 may arise from the maintenance of cellular metabolic status. Alternatively, the presence of PPARδ in astrocytes and the *in vivo* protective effects of GW0742 support an anti-inflammatory role for this ligand-activated transcription factor. It is possible that PPARδ agonism is neuroprotective via multiple modes of action and further work will be required to delineate the importance of each of these mechanisms to the neuroprotection afforded by PPARδ agonists.

## Disclosure statement

The authors declare that they have no conflict of interest.

## Figures and Tables

**Table 1:** Activity and receptor selectivity of GW0742 and GSK0660. The activity of GW0742 is expressed as the EC50 (μM) for this compound in a transactivation assay (Sznaidman et al., 2003), whilst the activity of GSK0660 is expressed as the IC50 (μM) in a **G**AL4 LBD chimera assay (Shearer et al., 2008)

	PPARα	PPARγ	PPARδ	Selectivity for PPARδ
GW0742 (EC_50_ μM)	1.1	2	0.001	>1000-fold
GSK0660 (IC_50_ μM)	>10	>10	0.155	>1000-fold

**Table 2:** Effects of genetic manipulation of PPARδ levels on striatal dopamine and DOPAC levels. No difference is seen between wild-type mice and their heterozygous littermates (null mice were not viable) in their sensitivity to MPTP toxicity as measured by reduction in dopamine and DOPAC levels. Data are mean ± SEM, *n* = 7 mice per group. ^∗∗∗^*p* < 0.001 compared to appropriate saline-treated group (Kruskal–Wallis test with Mann–Whitney U-post hoc tests; WT – wild-type, Het – heterozygous)

	Saline		MPTP	
	WT	Het	WT	Het
Dopamine (ng/mg wet tissue)	15.98 ± 1.87	19.06 ± 2.05	2.67 ± 0.44^∗∗∗^	1.73 ± 0.34^∗∗∗^
DOPAC (ng/mg wet tissue)	2.07 ± 0.32	1.85 ± 0.60	0.50 ± 0.06^∗∗∗^	1.12 ± 0.42

**Table 3:** Effects of intra-striatal infusion of GW0742 on striatal dopamine and DOPAC levels. No difference is seen in MPTP-induced reductions in dopamine and DOPAC levels between mice infused with GW0742 or those receiving vehicle (25% DMF in PBS – see Experimental procedures for details). Data are mean ± SEM, *n* = 3–5 mice per group. ^∗^*p* < 0.05 0; ^∗∗^*p* < 0.01 compared to appropriate saline-treated group (Kruskal–Wallis test with Mann–Whitney U-post hoc tests)

	Saline		MPTP	
	Vehicle	GW0742	Vehicle	GW0742
Dopamine (ng/mg wet tissue)	12.30 ± 1.69	10.82 ± 2.12	3.57 ± 0.43^∗∗^	3.53 ± 0.68^∗∗^
DOPAC (ng/mg wet tissue)	1.38 ± 0.18	1.32 ± 0.35	0.79 ± 0.06^∗^	0.63 ± 0.12

**Table 4:** Effects of intra-striatal infusion of GW0742 on striatal levels of MPP^+^. No differences were seen in striatal levels of MPP^+^ between mice infused with GW0742 or those receiving vehicle (25% DMF in PBS). Data are mean ± SEM, *n* = 2–5 mice per group

	Vehicle	GW0742
MPP^+^ (μg/g wet tissue)	11.54 ± 1.46	7.53 ± 1.39
